# Continuous Decolorization of Acid Blue 62 Solution in an Enzyme Membrane Reactor

**DOI:** 10.1007/s12010-015-1741-9

**Published:** 2015-08-06

**Authors:** Marcin Lewańczuk, Jolanta Bryjak

**Affiliations:** Faculty of Chemistry, Department of Bioorganic Chemistry, Wrocław University of Technology, Norwida 4/6, Wrocław, Poland

**Keywords:** Decolorization, Kinetic parameters, Laccase, Membrane bioreactor, Wastewater treatment

## Abstract

This paper focuses on using an enzyme membrane reactor (EMR) for the effective continuous decolorization of Acid Blue 62 (AB62). The following factors were considered for the effective use of *Cerrena unicolor* laccase immobilized in the EMR volume: the enzyme was stable in six successive runs in a batch reactor; no aeration was necessary; AB62 and the oxidized products were sorbed onto the membrane but were not rejected; and the enzyme was stable in the EMR system. It is obvious that any continuous process must be predictable, and thus, the objective was to verify the process model experimentally. For this reason, a proper isoenzyme kinetic equation was selected and the parameters were evaluated. The obtained kinetic parameters were used to plan processes and to verify their applicability to long-term AB62 decolorization, and a very good agreement between the calculated and the measured data was obtained. In the main designed continuous decolorization process, the conversion reached 98 % and was stable for 4 days. The membrane reactor with *C. unicolor* laccase appears to be very promising for AB62 decolorization.

## Introduction

In recent years, with the growing concern for clean environment, more attention has been paid to the pollutants generated by textile industry. Many dyes in the wastewater are toxic and potentially carcinogenic; hence, a great number of separation techniques have been developed for treatment of such effluents. The most commonly used are coagulation, flocculation, foam flotation, membrane filtration, and chemical processes [1, 2]. Physicochemical methods require a lot of energy, consume some chemicals, and can generate a substantial amount of precipitate that causes secondary pollution problems and requires additional processing [3]. Enzymatic or microbial dye degradation is regarded as an environmentally friendly, cost-competitive, and simple alternative to chemical decomposition processes [4].

Laccases (benzenediol:oxygen oxidoreductase, EC 1.10.3.2.) attract attention because they oxidize substrates without requiring expensive co-factors (mostly oxidoreductases) or H_2_O_2_ (peroxidases). These enzymes are reactive towards many phenolic and nonphenolic compounds and use dioxygen as the only electron acceptor. Hence, laccases can be used to treat effluents containing phenolic compounds from many industrial sectors, including textile one. These treatments are based on cross-coupling reactions, homo- or hetero-polymerization, or simple degradation [5]. Laccases are often used to decolor many dyes (see reviews [6, 7]) including anthraquinone ones, the most important subclass of carbonyl dyes. Unfortunately, most of these dyes are toxic, carcinogenic, or mutagenic and they are resistant towards light or oxidizing agents [8]. To remove them, several physicochemical [9–11] or biological [12–16] methods have been applied [8, 17, 18]. It seems that the interesting alternative for dye decolorization by biocatalyst is the application of a continuous system with a semipermeable membrane to separate products and an enzyme from a reaction mixture.

**Fig. 1:**
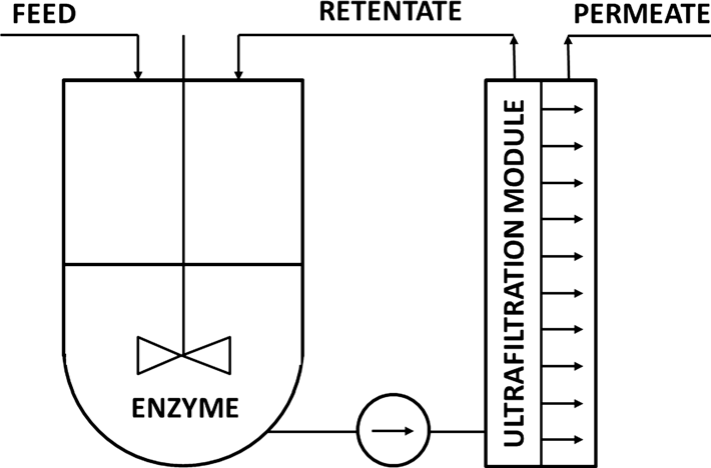
Set-up of a membrane reactor

An enzymatic membrane reactor (EMR) can operate in many different configurations that depend on the reaction type and enzyme properties [19]. The most frequently used mode is a membrane reactor with a soluble form of enzyme immobilized in the volume of reactor. The used ultrafiltration (UF) membrane with a 5–10-kDa-molecular-weight cutoff keeps an enzyme in reactor volume and does not allow it to be washed out (Fig. 1). EMRs have many advantages, among them one can point at the most important: (i) a membrane successfully stops enzymes; (ii) no loss of an enzyme is observed that is common in other immobilization methods; (iii) for unstable enzymes, the biocatalyst can be refreshed easily; (iv) homogeneous reactions are unaffected by the diffusion resistance; and (v) the system can be easily scaled-up [20]. On the other side, EMR applications have several limitations: (i) membrane fouling that can limit the filtration rate; (ii) the request for reactants to be soluble; (iii) the request for biocatalyst to be stable in the process conditions; (iv) the request for membrane to reject the enzyme completely but be permeable to product(s); and (v) the membrane material should be stable in the process conditions and cannot react with any mixture component [20].The use of laccase in EMR designated for dye decolorization seems to be a very promising but not so popular [21–23] approach. Moreover, the studies on decolorization modeling that take into account enzyme kinetics were rarely presented [24, 25].

The present investigation focuses on the decolorization of Acid Blue 62 (AB62) in EMR. AB62 was selected as the products of its oxidation by laccases are well known and they are less toxic than AB62 [26]. The process was performed using extracellular laccase from *Cerrena unicolor* because this biocatalyst can be produced without any inducers and its purification is cost effective [27]. Moreover, the decolorization of AB62 by this laccase does not require expensive mediators. A few physical and biological methods have been tested for AB62 decolorization [28–31], but to the best knowledge of the authors, there are no reports on the use of EMR. The present paper focuses on the enzyme stability, substrate/product sorption onto membrane, reaction kinetics, process modeling, and the model validation in long-term AB62 decolorization with soluble laccase immobilized in EMR.

## Materials and Methods

### Materials

AB62 was donated by Boruta-Zachem (Poland). Lowry’s reagent and 2,2-azino-bis(3-ethylbenzthiazoline-6-sulfonate) sodium salt (ABTS) were supplied by Sigma-Aldrich. Other reagents of analytical grade were purchased from POCH (Poland).

### Production of Laccase and Enzyme Activity Assay

The wood-rotting fungus *C. unicolor* (Bull. ex. Fr.) Murr, No. 139 was obtained from the culture collection of the Department of Biochemistry, University of Lublin (Poland). The microorganism cultivation, laccase production, and purification were performed according to methods described earlier [27].

**Fig. 2:**
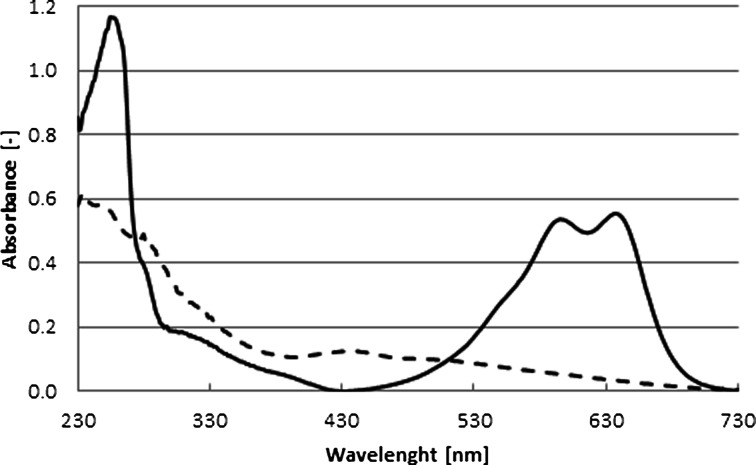
AB62 spectra (113.6 μM) before (*solid line*) and after (*dashed line*) decolorization with laccase

The progress of the dye conversion was followed using a spectrophotometric method in which the UV–Vis spectra from 220 to 800 nm were recorded for multiple time points. The change of absorbance (Fig. 2) was consistent with the data obtained by other researchers working with white-rot fungi [32–34] or purified laccases [26, 35]. The quantitative measurements of the substrate concentration were made at 637 nm (Fig. 2, solid line), in the region of insignificant absorbance of the reaction product (Fig. 2, dashed line). The substrate concentrations were calculated from the linear region of the calibration curve (*R*^2^ = 0.998). A spectrum of the product solution was recorded after 12 h incubation of the substrate with laccase (*R*^2^ = 0.993 for the calibration curve).

Laccase activity towards AB62 was measured as absorbance (637 nm) decrease in initial reaction time (linear dependence). One activity unit (U_AB62_) was defined as the amount of protein that caused a 2.5-μM decrease in the dye concentration per minute under reaction conditions (pH 5.3, 0.1 M phosphate-citrate buffer, 30 °C, 113.6 μM initial substrate concentration). The enzyme preparation used through this study had a specific activity of 11,000 U mg^−1^ protein. The protein concentration was assayed by Lowry’s method [36], using bovine serum albumin as a standard. Measurements of laccase activity and protein concentration were done in triplicate (±1.5 and ±2.6 %, respectively).

### Enzyme Activity and Stability in pH

The optimum pH was evaluated in the range of 4.7–6.5 at 30 °C. No color change was noted in control probes with the dye solutions of different pH without laccase. The value obtained in pH 5.3 was set as 100 %.

The pH stability was determined by incubating (30 °C) the sample at the given pH (ranging from 4.5 to 6.7) for 5 and 36 h. The pH of the solution was then adjusted to 5.3, and the preparation was left for 1 h to equilibrate and then its activity was measured (irreversible inactivation).The activity values measured just before incubation were set as 100 %.

All measurements were carried out in triplicate (±2.4 %).

### AB62 Decolorization in a Batch Reactor

AB62 solution (0.05 mL, 68.2 mM, final dye concentration 113.6 μM) was added to the enzyme solution in 0.1 M phosphate-citrate buffer (30 mL, 3 U_AB62_ mL^−1^). The absorbance decrease (637 nm, 30 °C) was measured to control the decolorization process. The reaction was monitored (20-s intervals) up to the substrate depletion (the stable absorbance level). The activity of laccase was calculated on the base of initial linear dependence of absorbance vs time. The value obtained in the first process was set as 100 %. Next, five consecutive portions of the substrate were added after substrate depletion.

The influence of oxygen consumption on the enzyme activity was checked by monitoring the oxygen concentration using a luminescent dissolved oxygen probe (LDO10101, Hach Lange).

### Membrane Start-Up

Studies were performed at 30 °C in a Labscale™ TFF System (Millipore, Bedford, USA), comprising a 500-mL reservoir stir base, retentate valve, pressure gauges, and a diaphragm pump, and with a Pellicon® XL module (Millipore) equipped with Biomax-5 membrane with 50 cm^2^ of filtration area. The Biomax-5 membrane is made of polyethersulfone, and its molecular weight cutoff is 5 kDa. Before the experiments, the UF unit with the Pellicon module was rinsed according to the supplier’s procedure. Pure water and 0.1 M phosphate-citrate buffer (pH 5.3) fluxes were measured prior to processing (1.49 10^−5^ ± 1.67 10^−7^ and 1.39 10^−5^ ± 1.00 10^−7^ m^3^ s^−1^ m^−2^, respectively, for transmembrane pressure (TMP) 0.137 MPa).

After completion of the experiments, the system was cleaned according to the manufacturer’s specification [27]. The cleaning process was kept running until the permeate flux was not lower than 95 % of its initial value.

### Studies on Membrane Separation Properties in the Presence of the Reactants

To test the separation/sorption properties of the membrane in the presence of reactants, the reservoir was filled with 50 mL of the tested solution, and the TMP was set at 0.137 MPa. The system was used in diafiltration mode. Permeate samples were collected at various times. After the diafiltration and retentate drainage, 50 mL of the buffer was added to the system. The permeate and retentate were recirculated without TMP for 30 min to wash the compounds that were loosely bound to the membrane. The retentate from this step was named the wash-out solution. The feed, wash-out, retentate, and permeate samples were analyzed.

The above procedure was applied using 50 mL of AB62 solution (113.6 μM) continuously fed into the membrane reactor (averaged permeate flux was 1.14 10^−5^ ± 2.33 10^−7^ m^3^ s^−1^ m^−2^). The absorbance was monitored at 637 nm in all samples. To test membrane in the presence of the reaction product, 2 L of the substrate solution was incubated at room temperature with laccase (2 U_AB62_ mL^−1^) for 12 h. Afterwards, the decolorized solution was used as a feed (averaged permeate flux was 9.33 10^−6^ ± 4.67 10^−7^ m^3^ s^−1^ m^−2^), and the absorbance was monitored at 490 nm.

To determine the amount of enzyme sorbed on all parts of the EMR system, the reservoir was filled with 50 mL of laccase solution (5 U mL^−1^). The retentate was recirculated for 180 min, and the permeate was turned back to retentate. The retentate samples were collected in specified time intervals and analyzed for laccase activity.

### Kinetic Studies

The initial rates of AB62 decolorization was measured by monitoring the absorbance decrease at 637 nm using 1.95 mL of buffered dye solution with AB62 concentrations changing from 12.5 to 250 μM and with 0.05 mL of laccase solution with a final protein concentration of 290 and 408 μg L^−1^ (3.2 and 4.5 U_AB62_ mL^−1^). All measurements were done at least in triplicate (±2.1 %). The Michaelis and rate constants were estimated by nonlinear regression by means of OriginPro 9 software.

### Continuous AB62 Decolorization in EMR and the Process Modeling

Continuous decolorization of AB62 was performed at 30 °C, using a buffered 113.6-μM solution of AB62 as feed. The reactor volume was filled with 50 mL of laccase solution (1.5 and 12 U_AB62_ mL^−1^). Decolorization was performed using selected permeate fluxes (5.67 10^−6^ and 4.67 10^−6^ m^3^ s^−1^ m^−2^) which were controlled by changing the TMP. The permeate samples (50 mL) were collected, and the absorbance was monitored at 637 nm. The steady-state conditions were confirmed by specifying the same absorbance values in permeate in three consecutive analysis in 0.5-h intervals.

A continuous operation in a stirred tank reactor was modeled for the following assumptions towards the system: the system runs in a continuous production mode; there is no concentration gradient with respect to the location (effluent concentrations are equal to reactor concentrations); the reaction rate (*r*) in the steady state is stable; the reactor is well-stirred; the reactor keeps constant volume (*Vr*) due to equal inlet and outlet fluxes ($\dot{V}$); and steady state is reached after start-up period (usually, it takes 4–5 times of the residence time, *τ*), where

1 $$ \tau =\frac{Vr}{\dot{V}} $$

Since at steady state the rate of accumulation is zero, the general component balance for a well-mixed continuous tank reactor has a following form [37]:

2 $$ 0=\dot{V}\bullet {C}_{\mathrm{S}0}-\dot{V}\bullet {C}_{\mathrm{S}}+{V}_r\bullet r $$

where *C*_S0_ and *C*_S_ are the initial substrate concentration and substrate concentration in a reactor at steady state, respectively.

The equation predicts that the reaction causes a depletion of substrate and that the reaction rate at steady state can be obtained from Eq. (3):

3 $$ r=-\frac{\left({C}_{\mathrm{S}0}-{C}_{\mathrm{S}}\right)}{\tau } $$

However, using the Michaelis-Menten approach, the reaction rate from Eq. (4) can be an equivalent of formula (3) at steady state:

4 $$ r=\frac{k_{\mathrm{cat}}\bullet {C}_{\mathrm{E}}\bullet {C}_{\mathrm{S}}}{K_{\mathrm{m}}+{C}_{\mathrm{S}}} $$

where *k*_cat_ is the rate constant, *C*_E_ is the enzyme concentration, and *K*_m_ is the Michaelis constant.

Hence, the model can be used to find the system dynamics and to calculate the steady-state values. It must be underlined that the modeling of a membrane-assisted continuous-stirred tank reactor is substantially the same with an assumption that a membrane is a physical barrier only, suitable for the retention of an enzyme.

## Results and Discussion

### Laccase Stability

To increase *C. unicolor* laccase potential applications, AB62 decolorization was performed without any natural or synthetic mediators, and the enzyme preparation was only partly purified by means of micro- and ultrafiltration [27].

**Fig. 3:**
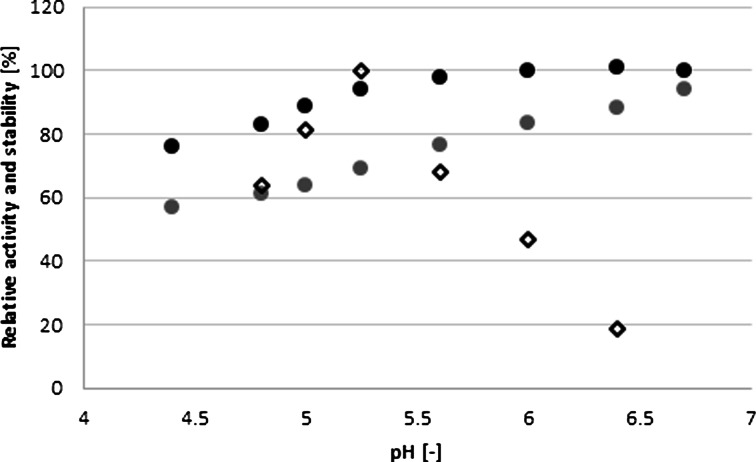
Effect of pH on AB 62 decolorization by laccase (*white diamond*) and on the enzyme stability after 5 (*black circle*) and 36 h (*gray circle*) of incubation in the buffer

The most important process parameters are influence of pH and temperature on enzyme activity and enzyme conformational stability. In the case of laccase, its temperature profile and thermal stability were tested previously [38], and it was shown that 30 °C is the very good compromise between the reactivity and the stability. Taking into account pH influence, it must be tested in the presence of any given substrate. As seen from Fig. 3, the highest rate of AB62 decolorization is noted in pH 5.3, whereas stability is perfect in a more neutral pH but at the cost of reactivity. Thus, pH 5.3 was selected that provides the highest activity and satisfactory stability. Interestingly, Michniewicz et al. [35] showed a maximum AB62 decolorization efficiency around pH 3.5 in the case of laccase from *C. unicolor* but originated from different strains (137).

**Fig. 4:**
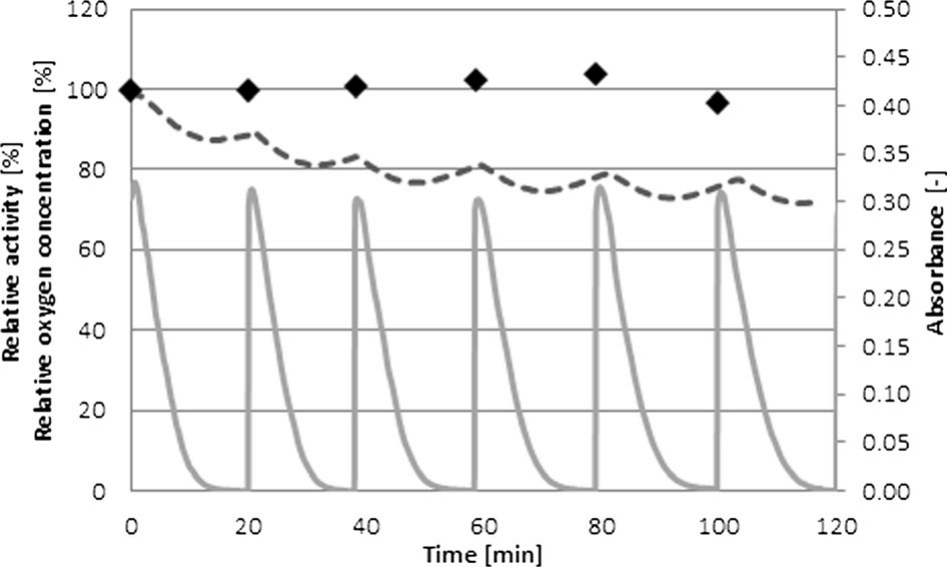
Repeated batch decolorization (637 nm) of AB62 (113.6 μM) using native laccase (3 U_AB62_ mL^−1^) at pH 5.3 and 30 °C. *Solid line* absorbance after baseline correction, *black diamond* laccase activity, *dashed line* oxygen concentration

The effective use of laccase immobilized in the EMR volume for dye decolorization depends on sufficient enzyme stability in the processing conditions. To test the *C. unicolor* laccase stability in the presence of the substrate and reaction products, six successive batch processes were run in a well-stirred reactor. A fresh substrate was added after the depletion of the previous dose that was manifested as a constant minimal absorbance level (Fig. 4, gray solid line). During these measurements, the laccase activity remained almost the same, and thus, one can conclude that the enzyme is not inactivated by the substrate and/or reaction products and can be used in EMR.

Laccases catalyze the oxidation of a wide range of substrates, and this phenomenon is coupled to the reduction of molecular oxygen to water. Thus, the rate of many reactions can be limited by oxygen consumption. For that reason, the oxygen concentration was monitored (Fig. 4, dotted line) in the process, and the collected results indicated that oxygen consumption should not significantly affect AB62 decolorization. This finding reduces the request for an additional aeration of the reaction mixture for planned processes in EMR.

### Sorption of Laccase, AB62, and Products on the Membrane in the EMR Set-Up

**Fig. 5:**
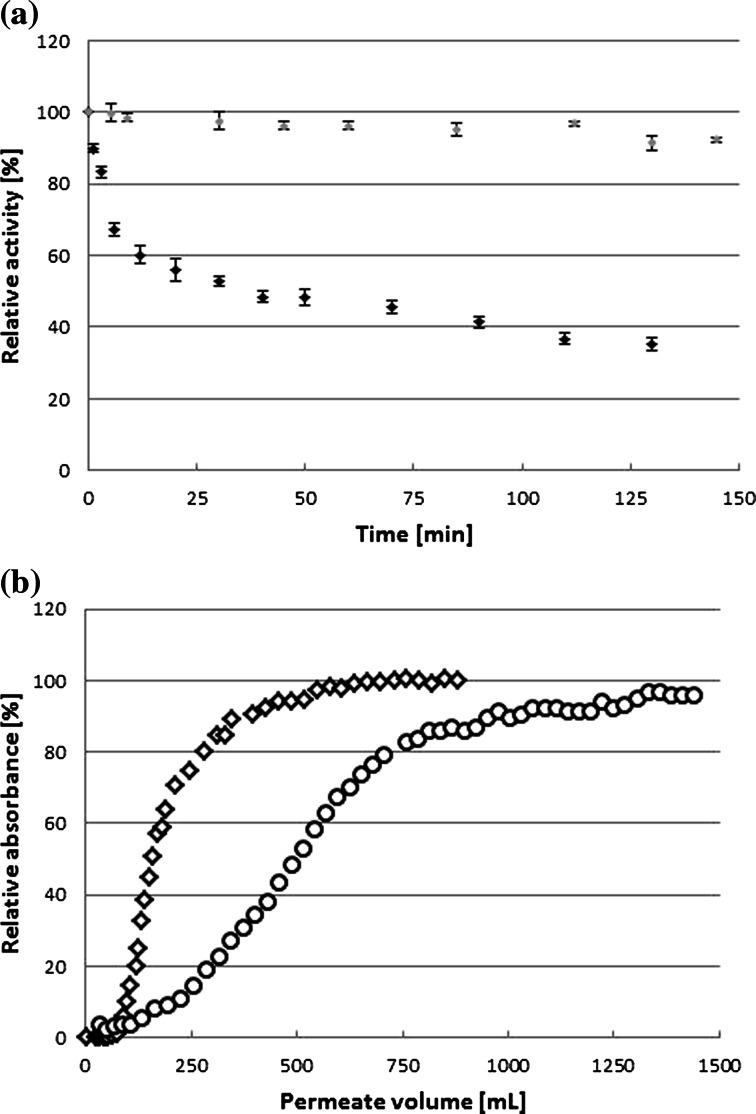
**a** Laccase activity in the retentate during the retentate and permeate recirculation in the EMR (*black diamond*) and during incubation in a stirred tank reactor (*black circle*). Conditions: pH 5.3; 30 °C; 100 rpm; 0.137 MPa, laccase concentration of 5 U mL^−1^ (453 μg L^−1^). **b** Absorbance in the permeate during membrane tests using AB62 (*white diamond*; 637 nm) or oxidized AB62 (*white circle*; 490 nm) relative to the feed solution. Tests were performed in diafiltration mode at a transmembrane pressure of 0.137 MPa, *τ* = 13.9 min, pH 5.3, 30 °C

The complete retention of laccase by the polyethersulfone membrane with a molecular weight cutoff of 5 kDa was demonstrated in our previous work [39]. The objective of the present work was to quantify the formation of sorption and/or polarization layer of the enzyme, substrate, and products. In the case of laccase, the activity in the retentate was analyzed in a system, in which the permeate was returned into retentate (Fig. 5a, black points). The decrease of enzyme activity within the first 15 min of the process was observed. This decrease can be attributed to the sorption of protein molecules onto either the system surface or the membrane. Unfortunately, the highly active preparation made it difficult to assay the protein concentration, and the evaluation of the enzyme activity was the only possible way to monitor the sorption phenomenon. In such situation, laccase inactivation process could not be excluded. Therefore, the change in laccase activity with time in a stirred batch reactor was evaluated (Fig. 5a, gray points) and used as a control experiment. The perfect laccase stability in the batch test suggested that the loss of enzyme activity in the EMR was caused mostly by sorption than by the enzyme inactivation. Similar results were obtained previously when a 5-kDa membrane was tested for laccase retention in a continuous mode [39]. In that case, the loss of approximately 18 % of the enzyme activity was noted during the first 20 min of the pre-conditioning, and after that, the activity balance (95.4 %; the sum of total activity in retentate, permeate, and wash-out solution in respect to initial activity) showed almost no activity loss.

Sorption of the enzyme on the membrane surface can be regarded as a serious drawback. Most likely, part of the protein could adsorb in an unfavorable orientation with the active center of the enzyme directed towards the membrane surface, blocking access of substrate [40]. However, sorption of the enzyme, that is still active, onto the membrane can cause a local enzyme concentration increase, making it possible for the substrate to be converted to the product just before leaving a reactor space. However, it was expected that the enzyme sorption onto the membrane could be responsible for lower substrate conversion.

It is obvious that the membrane used in EMR must be permeable to the product; in contrast, this requirement is not as obvious for the substrate. Taking these requests into account, the possibility for rejection of AB62 or its oxidized product was tested (Fig. 5b). Surprisingly, at the very beginning of the test, neither reactant was visible in the permeate, although 50 mL of substrate or reaction product was poured into the EMR system and then reactants were continuously pumped into the reactor. This finding evidenced the strong sorption of both compounds onto the membrane. It was noted that, up to the moment of membrane breakthrough, the reaction volume was exchanged once (substrate) and three times (products). Moreover, the steady-state conditions were obtained after 16 (substrate) or 29 (products) volume exchanges. Thus, it was expected that the main problem could be to stabilize the concentrations of all reactants (enzyme, AB62, products) in the reaction device. Sorption of the product is a bottleneck in achieving steady-state conditions in the considered continuous process. Thus, the reaction rate could be stable after 6–7 h of the real reaction, which is equivalent to 30 times of the residence time. Fortunately, the membrane was permeable to AB62 and its oxidized products, although substantially more volume exchanges are required to obtain a steady-state regime than in conventional continuous systems (usually 4–5 times of the residence time).

### Kinetics of AB62 Decolorization with Laccase

**Fig. 6:**
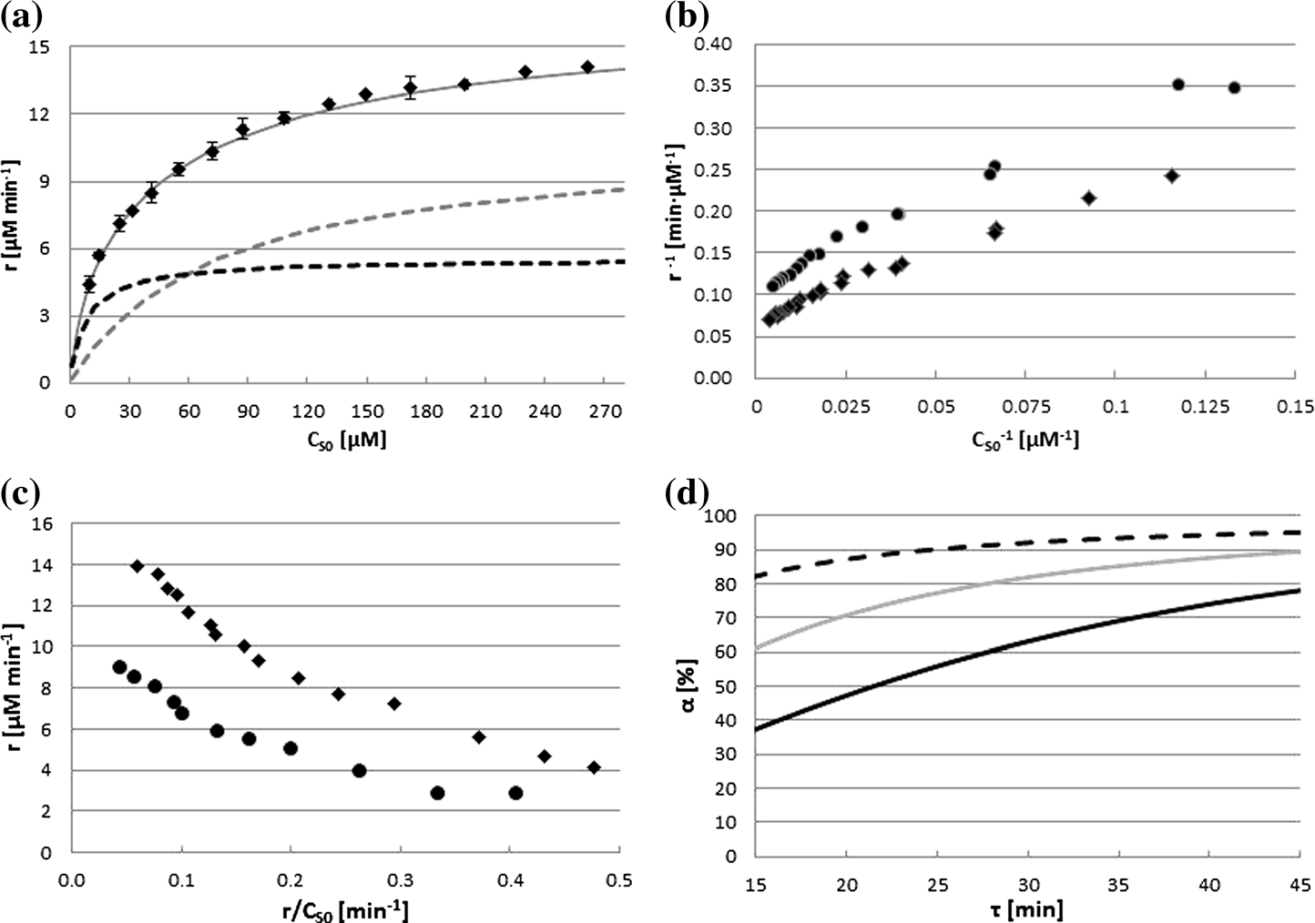
Example of the effect of initial substrate concentration (*C*_S0_) on the initial reaction rate (*r*) (**a**), Lineweaver-Burk plot (**b**), Eadie-Hofstee plot (**c**), and modeled (Eq. 5) variations of the conversion degree (*α*) with residence time (*τ*) (**d**). The *solid line* in **a** demonstrates the accuracy of the fitting of the experimental data to the isoenzyme model (Eq. 5) and the estimated parameters (Table 1). The *dotted lines* represent the hypothetical modeling for isoenzyme 1 (*gray*) and 2 (*black*). Reaction conditions: pH 5.3; 28 °C; enzyme concentration in **a** 3.2 U_AB62_ mL^−1^ (290 μg L^−1^); in **b** and **c**(*black diamond*) 4.5 U_AB62_ mL^−1^ (408 μg L^−1^) and (*white diamond*) 3.2 U_AB62_ mL^−1^ (290 μg L^−1^); in **d** 1.5 U_AB62_ mL^−1^—*black solid line*; 4.5 U_AB62_ mL^−1^—*gray solid line*; 12.0 U_AB62_ mL^−1^—*black dashed line*, (136, 408, 1088 μg L^−1^, respectively)

Prediction of the dye conversion in the continuous reactor is a critical issue. For this reason, a proper kinetic equation has to be selected and its parameters should be estimated. An enzyme immobilized in the volume of a membrane reactor provides a homogenous reaction system; therefore, kinetic parameters can be found using data from a batch reactor with a native enzyme. The interactions between AB62 and the enzyme were defined by the commonly used Eq. (3) (Fig. 6a; experimental points). However, in the case of AB62 decolorization, some deviations from a simple 1/r versus 1/C_S0_ dependence (Fig. 6b) were observed. These discrepancies are better visualized by the Eadie-Hofstee plot (Fig. 6c). Such behavior is attributed to the presence of multiple forms of the same enzyme (isoenzymes) that act on the same substrate but with different maximum reaction rates (${V}_{\max (i)\ }\Big)$ and/or Michaelis constants (${K}_{\mathrm{m}(i)}\Big)$ [40]. The obtained results confirmed data obtained by other authors, who reported that the enzyme preparations from *C. unicolor* consist of two isoforms of laccase [35, 41]. In this case, the reaction rate at any substrate concentration is the sum of the velocities contributed by each isoform and is given by Eq. (5).

5 $$ r={r}_1+{r}_2=\frac{V_{\max 1}\bullet {C}_{\mathrm{S}0}}{K_{\mathrm{m}1}+{C}_{\mathrm{S}0}}+\frac{V_{\max 2}\bullet {C}_{\mathrm{S}0}}{K_{\mathrm{m}2}+{C}_{\mathrm{S}0}} $$

where the indexes 1 and 2 indicate the isoform number and

6 $$ {V}_{\max (i)}={C}_{\mathrm{E}(i)}\bullet {k}_{\mathrm{cat}(i)} $$

where *C*_E_ is the enzyme concentration.

Assuming that

7 $$ {\mathrm{C}}_{\mathrm{E}}={C}_{\mathrm{E}1}+{C}_{\mathrm{E}2} $$

where *C*_E1_ and *C*_E2_ are the concentration of enzyme isoforms 1 and 2, respectively; Eq. (5) has a form:

8 $$ r=\frac{k_{\mathrm{cat}1}\bullet {C}_{\mathrm{E}1}\bullet {C}_{\mathrm{S}0}}{K_{\mathrm{m}1}+{C}_{\mathrm{S}0}}+\frac{k_{\mathrm{cat}2}\bullet \left({C}_{\mathrm{E}}-{C}_{\mathrm{E}1}\right)\bullet {C}_{\mathrm{S}0}}{K_{\mathrm{m}2}+{C}_{\mathrm{S}0}} $$

Equation (5) allowed to estimate four kinetic parameters by means of nonlinear regression (Table 1). As seen, the *K*_m_ values for two different enzyme concentrations were similar what confirmed experimental accuracy. Moreover, fitting of the experimental points by the model (Fig. 6a) was very good. However, a simultaneous trial to determine all kinetic constants, *K*_m(*i*),_*k*_cat(*i*)_ and the concentration of isoform 1 (*C*_E1_ in Eq. 7) failed due to the large number of parameters to be determined (Eq. 8). Hence, to limit the number of parameters to be estimated, the concentration of laccase isoforms was approximated from the ratio of the reaction rates of the isoforms (*r*_1_:*r*_2_) for the infinite substrate concentration, based on the *V*_max(*i*)_ and *K*_m(*i*)_ parameters (Table 1). The calculated ratio was 1.85 and was independent of *C*_E_. Moreover, this value was reasonably similar to that presented by Michniewicz et al. [35] (1.48) what entitled us to calculate protein concentrations of both enzyme forms. Finally, the values of *k*_cat1_ and *k*_cat2_ were estimated using nonlinear regression with fixed Michaelis constants. The number of parameters that had to be determined (Table 1) in this operation was reduced to two. The very good confidence intervals demonstrated the mathematical correctness of the estimated constants.

**Table 1:** Kinetic parameters of AB62 decolorization by *C. unicolor* laccase

Parameter	Enzyme concentration (μg L^−1^)	
	290	408
*V*_max1_ (μM min^−1^)	7.37 ± 1.07	11.01 ± 2.92
*K*_m1_ (μM)	72.90 ± 28.42	67.99 ± 32.87
*V*_max2_ (μM min^−1^)	3.67 ± 1.45	5.14 ± 3.46
*K*_m2_ (μM)	5.64 ± 3.40	6.62 ± 2.46
*k*_cat1_ (min^−1^)	0.0331 ± 0.0009	0.0375 ± 0.0011
*k*_cat2_ (min^−1^)	0.0317 ± 0.0008	0.0351 ± 0.0010

Despite the same substrate, comparison of obtained values with those presented by other authors was very difficult (Table 2) and caused by different origins of laccases and/or their purity and process parameters (pH, temperature). Even in the case of *C. unicolor* isoforms, a significant difference between the *k*_cat_ constants was noted (rows 1 and 2 in Table 2), but this could be caused by the difference with the pH values used here and in the work by Michniewicz et al. [35], and by the enzyme purity. On the other hand, the *K*m values obtained for both isoforms here and in [35] appeared to be quite similar. Hence, it seems that the *C. unicolor* isoforms differ in enzyme-substrate affinities, whereas the reaction rate constants for both isoenzymes do not differ too much.

**Table 2:** Kinetic constants of the Michaelis-Menten equation for AB62 decolorization with laccases from different sources

Microorganism	pH (−)	*T* (°C)	*K*_m_ (μM)	*k*_cat_ (s^−1^)	Reference
*Cerrena unicolor* No. 139	5.3	30	6.22	38.5^a^	[here]
			74.11	41.8^a^	
*Cerrena unicolor* No. 137	3.5	25	42	366	[35]
			131	267	
*Trametes versicolor*	5.5	30	130	100	[42]
*Trametes versicolor*	4.5	na	305	–	[43]
*Trametes versicolor* ATCC 32745	4.5	na	302	1373	[43]
			204	898	
*Phoma sp*. UHH 5-1-03	4.0	RT	284 ^b^	32.2 ^b^	[44]
*Phoma sp.* UHH 5-1-03	4.5	na	194	34.1	[43]
*Bacillus subtilis*	4.5	na	165	3.1	[43]
*Pycnoporus sanguineus* MUCL 41582	4.5	na	472	35.7	[43]
Roglyr Lite 1540^c^	4.7	25	90	45.5	[45]

^a^Values recalculated according to units used by other researchers

^b^Parameters derived from Hill’s equation

^c^Commercial product (Rotta Manheim)

*RT* room temperature, *na* not available

Finally, the possibility of product inhibition was examined. The initial reaction rates were measured in mixtures using a constant substrate concentration and product concentrations that ranged from 0 to 150 μg L^−1^. The laccase activity remained unchanged (3.14 ± 0.045 U_AB62_ mL^−1^). This finding led to the conclusion that there was no enzyme inhibition by the products of the AB62 oxidation and that the Eq. (8) should be sufficient to predict the variations in reaction rate with substrate concentrations for selected laccase concentration in a continuous-mode membrane reactor.

### Validation of the Kinetic Model in Continuous Decolorization in EMR

The obtained kinetic parameters were used to plan processes and to verify their applicability to long-term AB62 decolorization. First, the value of the residence time (*τ*), that would allow an assumed conversion degree (*α*) for specified initial substrate and enzyme concentrations (Eq. 8), was calculated from Eq. (9).

9 $$ r=\frac{\alpha \bullet {C}_{\mathrm{S}0}}{\tau }=\frac{k_{\mathrm{cat}1}\bullet {C}_{\mathrm{E}1}\bullet {C}_{\mathrm{S}0}\bullet \left(1-\alpha \right)}{K_{\mathrm{m}1}+\bullet {C}_{\mathrm{S}0}\bullet \left(1-\alpha \right)}+\frac{k_{\mathrm{cat}2}\bullet {C}_{\mathrm{E}2}\bullet {C}_{\mathrm{S}0}\bullet \left(1-\alpha \right)}{K_{\mathrm{m}2}+\bullet {C}_{\mathrm{S}0}\bullet \left(1-\alpha \right)} $$

**Fig. 7:**
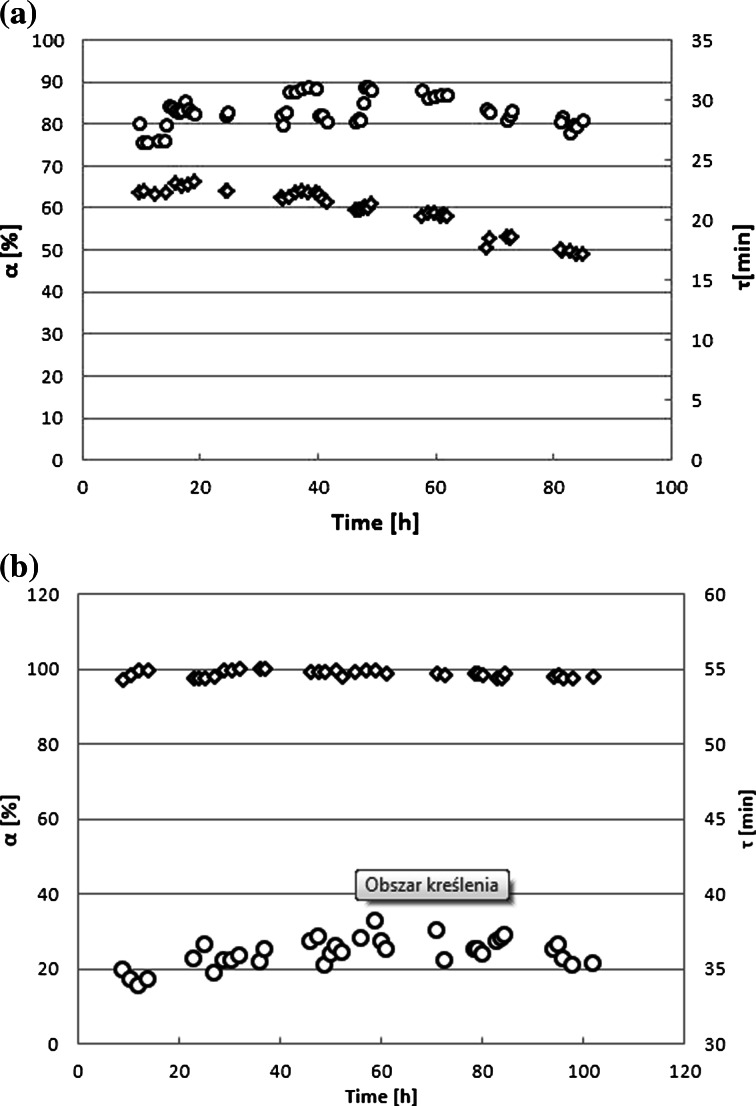
AB62 decolorization with laccase immobilized in the EMR volume. *White circle* residence time; *white diamond* conversion degree. Data obtained in the first 7-h period were removed (unsteady-state conditions). Reaction conditions: AB62 concentration of 113.6 μM in the feed; reactor volume of 50 mL; pH 5.3; 30 °C; enzyme concentration 1.5 U_AB62_ mL^−1^ (**a**) and 12 U_AB62_ mL^−1^ (**b**)

Figure 6d represents examples of the modeled relationship of *α* vs. *τ* for selected laccase concentrations with the process parameters shown in Table 3. In the first continuous process (Fig. 7a), a very low laccase activity and moderate residence time were applied. In such conditions, the substrate conversion should be not high, and any deviations from the expected values could be detected easily.

**Table 3:** Verification of AB62 decolorization with laccase immobilized in the volume of the membrane reactor

C_E_ (μg L^−1^)	Parameter (−)	Obtained values		
		Assumed	Experimental	Calculated (Eq. 9)
136	*τ* (min)	30	29.9 ± 1.4	29.9
	*α* (%)	65	60.0 ± 5.3	64.5
1088	*τ* (min)	36	36.7 ± 1.6	34.2
	*α* (%)	94	98.3 ± 0.9	93.2

The aim of the first long-term process was to evaluate the applicability of the AB62 decolorization equation for the prediction of substrate conversion. This model did not take into account factors such as reagent sorption on the membrane surface, deactivation, and/or unfavorable enzyme orientation after deposition on the membrane material. Table 3 (experimental and model values) shows that differences observed are statistically insignificant, although slightly lower conversion can be obtained in the experimental process. Most likely, the adsorption of laccase onto the membrane and/or the enzyme inactivation caused a decrease of the reaction rate. The entire process was conducted for 80 h, during which the permeate flux decay was compensated by an increase of the transmembrane pressure (from 0.137 up to 0.188 MPa). The stable residence time (Fig. 7a) allowed us to conclude that the conversion gradient in permeate was originated from the laccase activity loss. Plotting the reaction rate values with time and using the semi-logarithm relationship (data not shown), it was found that the loss of laccase activity obeyed the first-order kinetics with a rate constant of 0.00367 ± 0.00006 h^−1^.

The success in deriving the kinetic model to the prediction of the continuous process enabled us to run the next operation, in which 94 % conversion was assumed. The permeate flux was set at a level for which it was easy to control the residence time (see Table 3). To achieve this goal, the enzyme concentration was increased eight times. Figure 7b shows that residence time was stable and maintained by the increase of transmembrane pressure (from 0.137 up to 0.199 MPa); however, the ability to control the flux was limited at longer times. Interestingly, the laccase activity loss, that was visible in the first run of AB62 decolorization (Fig. 7a), was not observed in this case. Probably, the higher enzyme concentration increased the conformational stability of the protein what is a well-known phenomenon [46]. Therefore, a stable and very high substrate conversion (98.3 ± 0.866 %) was achieved for more than 100 h continuous process. Comparing assumed substrate conversion and obtained in the experiment, significant statistical differences are noted (expected yield was of 94.8 % of experimental yield). However, expected and experimental *τ* values differ similarly (93.2 %). Thus, although statistical differences are significant, they are proportional and that indicates some obstacles connected with polarization layer formed on the membrane.

Finally, we can compare our results with that obtained by other methods of AB62 decolorization [28–31]. In all the cases, substrate conversions were high, over 90 %. However, electrochemical degradation can be regarded as an energy-consuming process that demands long optimization of process conditions [1, 29]. In the case of absorption [28, 30], the main problem arises in the utilization of highly concentrated pollutant. Considering photocatalytic degradation over CuO–SnO_2_ nanocomposite [31], a potential practical problem must be underlined; sunlight dependence excluded continuous process. Thus, laccase immobilized in the membrane reactor seems to be an energy-saving and environmentally safe solution for AB62 continuous decolorization.

## Conclusions

It is obvious that any continuous process must be predictable. For that reason, isoenzyme model was applied, and on the base of derived parameters and the general equation for a continuous stirred tank reactor, the expected AB62 conversions were estimated and experimentally verified in the continuous processes. In the final process, the enzyme concentration and residence time were selected to obtain 94.0 % substrate conversion. In this process, the conversion reached 98.3 % and was stable for 4 days. Thus, the membrane reactor with *C. unicolor* laccase seems to be a very promising tool for AB62 decolorization.
